# Timing of The First Zygotic Cleavage Affects Post-Vitrification Viability of Murine Embryos Produced *In Vivo*

**DOI:** 10.22074/ijfs.2015.4243

**Published:** 2015-07-27

**Authors:** Wan-Hafizah Wan Jusof, Nor-Ashikin Mohamed Noor Khan, Mohd Hamim Rajikin, Nuraliza Abdul Satar, Mohd-Fazirul Mustafa, Norhazlin Jusoh, Razif Dasiman

**Affiliations:** 1 Institute of Medical Molecular Biotechnology, Faculty of Medicine, Universiti Teknologi MARA, Sungai Buloh, Selangor, Malaysia; 2 Maternofetal and Embryo (MatE) Research Group, Health and Wellbeing CoRe, Universiti Teknologi MARA, Shah Alam, Selangor, Malaysia; 3 Faculty of Medicine, Universiti Teknologi MARA, Sungai Buloh, Selangor, Malaysia; 4 Faculty of Health Sciences, Universiti Teknologi MARA, Bandar Puncak Alam, Selangor, Malaysia

**Keywords:** Vitrification, Early Cleavage, Mouse Embryos

## Abstract

**Background:**

Timing of the first zygotic cleavage is an accurate predictor of embryo quality. Embryos that cleaved early (EC) have been shown to exhibit higher develop- mental viability compared to those that cleaved at a later period (LC). However, the vi- ability of EC embryos in comparison to LC embryos after vitrification is unknown. The present study aims to investigate the post-vitrification developmental viability of murine EC versus LC embryos.

**Materials and Methods:**

In this experimental study, female ICR mice (6-8 weeks old) were superovulated and cohabited with fertile males for 24 hours. Afterwards, their ovi- ducts were excised and embryos harvested. Embryos at the 2-cell stage were catego- rized as EC embryos, while zygotes with two pronuclei were categorized as LC embryos. Embryos were cultured in M16 medium supplemented with 3% bovine serum albumin (BSA) in a humidified 5% CO_2_atmosphere. Control embryos were cultured until the blastocyst stage without vitrification. Experimental embryos at the 2-cell stage were vitri- fied for one hour using 40% v/v ethylene glycol, 18% w/v Ficoll-70 and 0.5 M sucrose as the cryoprotectant. We recorded the numbers of surviving embryos from the control and experimental groups and their development until the blastocyst stage. Results were analyzed using the chi-square test.

**Results:**

A significantly higher proportion of EC embryos (96.7%) from the control group developed to the blastocyst stage compared with LC embryos (57.5%, P<0.0001). Similarly, in the experimental group, a significantly higher percentage of vitrified EC embryos (69.4%) reached the blastocyst stage compared to vitrified LC embryos (27.1%, P<0.0001).

**Conclusion:**

Vitrified EC embryos are more vitrification tolerant than LC embryos. Prese- lection of EC embryos may be used as a tool for selection of embryos that exhibit higher developmental competence after vitrification.

## Introduction

One of the major problems in assisted reproductive technology (ART) is identification of good quality embryos. This is very important because the number of transferred embryos has to be low (preferably one embryo) in order to reduce the incidence of multiple pregnancies in women.

Multiple transfers can increase the risk of postpartum hemorrhage, pregnancy induced hypertension and anemia, as well as maternal mortality. It is also associated with higher rates of pre-term delivery, low birth weight, neonatal morbidity and infant death (1). To avoid complications associated with multiple pregnancies, transfer of a single embryo is highly recommended. However, the major concern among practitioners is the reduced success rate after transfer of a single embryo. Hence, if a good quality embryo can be identified and used in single embryo transfers, the likelihood of pregnancy will be increased.

Morphological evaluation has been the common method used in assessment of embryo quality (2). This method requires observational skills and may also be subjective, leading to inconsistencies. There can be a bias in the assessment of an embryo between different evaluators and also between different laboratories or clinics.

Timing of the first zygotic cleavage has been used as an alternative predictor for embryo quality in humans (3-5). Embryos that cleave early were proven to develop into good quality embryos with higher developmental viability compared to their late cleaving (LC) counterparts (5-8). Despite the fact that cryopreservation of human embryos is a common method in ART procedures, comparative studies on the viability of early cleaving (EC) and LC embryos after cryopreservation are lacking.

This study was therefore conducted to compare the cryotolerance of EC and LC murine embryos by evaluating their developmental viability after vitrification.

## Materials and Methods

### Embryo collection

In this experimental study, a total of 26 female ICR mice, aged 6-8 weeks were superovulated by intraperitoneal (i.p.) injections of 5 IU pregnant mare serum gonadotropin (PMSG, Folligon, Intervet International B.V, Holland) followed 48 hours later by an i.p. injection of 5 IU human chorionic gonadotropin (hCG, Chorulon, Intervet International B.V, Holland). Females were subsequently mated with male mice of the same strain at a ratio of 1:1. The morning after mating, females were checked for the presence of a vaginal plug. After 28 hours from hCG administration, oviducts from the plugged female mice were excised and embryos flushed out in M2 medium (Sigma, USA). Embryos were assessed under an inverted microscope (Leica, Germany). One-cell embryos with 2 pronuclei and embryos at the 2-cell stage were considered fertilized. All procedures that involved animals were approved by the Animal Care and Use Committee (ACUC), UiTM (ACUC-7/11).

### Timing of the first zygotic cleavage

Embryos were divided into two groups - EC and LC according to the timing of the first zygotic cleavage. Embryos that displayed 2-cells at 28-30 hours post-hCG administration were categorized as EC embryos while zygotes that contained a second polar body and two pronuclei were categorized as LC embryos. Embryos were cultured in 50 μl drops of the M16 culture medium (Sigma, USA) plus 3% bovine serum albumin (BSA, Sigma, USA) overlaid with mineral oil (Sigma, USA) in an atmosphere of 5% O_2_, 5% CO_2_ and 90% N_2_.

Control group embryos were cultured until the blastocyst stage without being subjected to vitrification. Embryo viability was assessed by embryo development in culture until the blastocyst stage. The developing embryos were observed under an inverted microscope every 24 hours. The developmental kinetics for a normal developing murine embryo is as follows: ≥2-cell at 24 hours, ≥4-cell at 48 hours, at least morulae at 72 hours and expanded blastocysts at 96 hours (9). Experimental embryos were vitrified at the 2-cell stage and subjected to culture after warming.

### Vitrification

The vitrification method used in this study was developed by Kasai et al. (10) and described in detail by Shaw and Kasai (11). The cryopreservation solution consisted of M2 medium with 40% v/v ethylene glycol, 18% w/v ficoll 70 and 0.5 M sucrose (EFS40). A Styrofoam box with a lid was used as a cooling container. This box was filled with at least 5 cm liquid nitrogen and precooled for 30 minutes before use. A Styrofoam boat with a thickness of 1 cm was made with grooves for holding straws.

A total of 30 μl of EFS40 solution were aspirated by connecting the straw to a pipett. This was followed by aspirating a total of 10 embryos in 10 μl of M2 and another 30 μl of EFS40 solution into the straw. Then, the straw was sealed at the open end using polyvinyl alcohol (PVA). The straw was then placed in the horizontal position and embryos equilibrated with the cryoprotectant for 1 minute at room temperature. Once ready, the straw was transferred to a Styrofoam boat and left floating on liquid nitrogen vapor for five minutes before being immersed in liquid nitrogen for 1 hour.

### Warming

The straw was transferred from the liquid nitrogen and placed for 5 minutes on the Styrofoam boat inside a Styrofoam box that contained liquid nitrogen. Using a pair of forceps, the straws were lifted from the boat and held in air for 10 seconds before being immersed in a 37˚C water bath for 10 seconds. The contents of the straws were expelled into M2 medium that contained 0.5 M sucrose in a culture dish. After 3 minutes, the culture dish was agitated gently to mix the dilution and cryoprotectant solutions. After a 5-minute incubation in 0.5 M sucrose, embryos were transferred to a new petri dish that contained M2 medium.

### Assessment of survival rate of vitrified embryos

**Fig.1:**
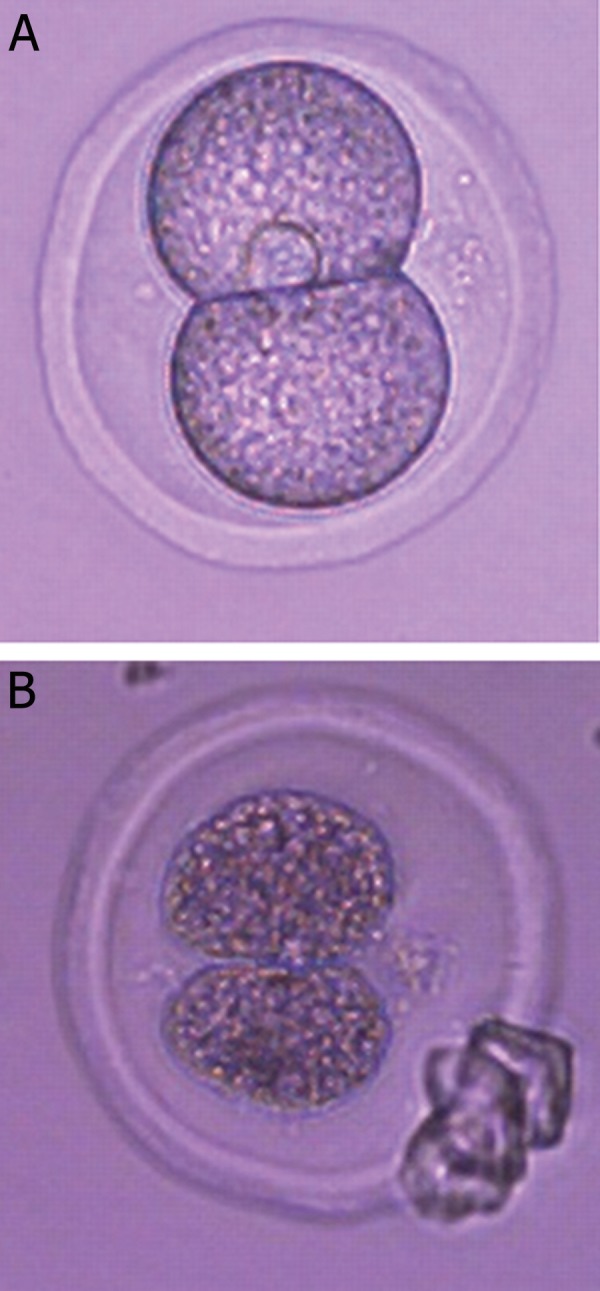
Morphology of murine embryos after vitrification (A) intact (B) degenerated.

The post-vitrification survival rate of embryos was assessed by evaluation of their morphology under an inverted microscope followed by development *in vitro* until the blastocyst stage. Embryos with intact blastomeres and zona pellucida after warming were classified as surviving embryos (Fig.1A). Degenerated embryos were discarded (Fig.1B).

### Embryo culture

Thawed embryos that had proper morphology were then transferred into fresh 50 μl droplets of the M16 medium plus 3% BSA, overlaid with mineral oil and cultured in an atmosphere of 5% O_2_, 5% CO_2_ and 90% N_2_.

Embryo viability was assessed by embryo development in culture until the blastocyst stage. Embryo development was monitored every 24 hours under an inverted microscope. The developmental kinetics for normal developing murine embryos after vitrification is as follows: ≥4-cells at 24 hours, at least morulae 48 hours and expanded blastocysts at 72 hours.

### Statistical analysis

Statistical analysis was performed using the SPSS software for Windows version 19.0.1 (Statistical Package for the Social Sciences, Inc., USA). Embryonic survival rates subsequent to vitrification and thawing, the developmental rates of embryos at different stages, and the blastocyst formation rates were determined and reported as percentages. The difference between the two groups (EC and LC embryos) was analyzed using the chi-square test. A P value of less than 0.05 was considered statistically significant.

## Results

### Control group (non-vitrified)

**Fig.2:**
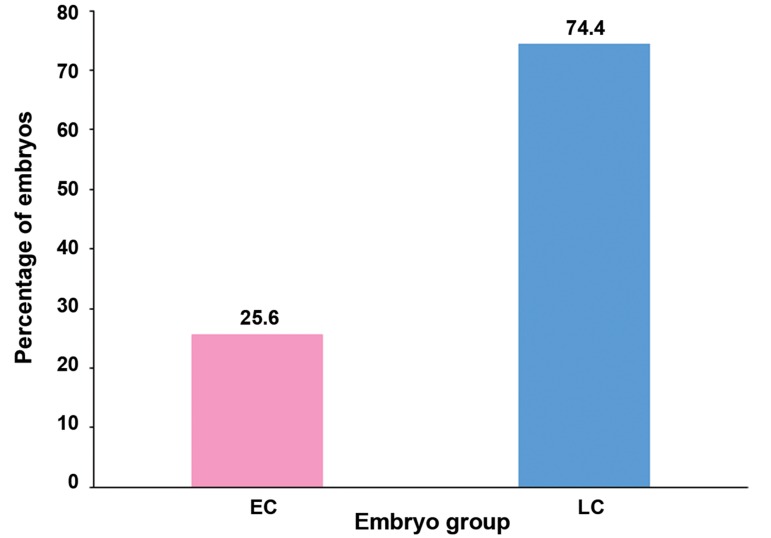
Percentage of early cleaving (EC) and late cleaving (LC) embryos from the control group (non-vitrified).

There were 234 embryos in the control group (non-vitrified). Out of this number, 60 (25.6%) were EC embryos while the other 174 (74.4%) were LC embryos (Fig.2). The developmental potential of both EC and LC embryos is summarized in table 1.

**Table 1:** Developmental kinetics of control early cleaving (EC) and late cleaving (LC) embryos following *in vitro* culture for 96 hours


Embryo groups	Number of embryos (%)		
	2-cell stage (24 hivc)	4-cell stage (48 hivc) Morula stage (72 hivc)	Blastocyst stage (96 hivc)

EC	60 (100)	59 (98.3) ** 59 (98.3) **	58 (96.7) **
LC	174 (100)	125 (71.8) 118 (67.8)	106 (60.9)


**; P<0.0001 versus LC embryos and hivc; Hours of *in vitro* culture.

There was a significantly higher percentage of developing EC embryos compared to LC embryos at 48, 72 and 96 hours (P<0.0001). After 48 and 72 hours of culture, 98.3% of EC embryos reached the 4-cell and morula stages. For LC embryos, the percentage was significantly lower than EC embryos after 48 and 72 hours of culture where 71.8% reached the 4-cell stage and 67.8% reached the morula stage (P<0.0001). After 96 hours of culture, the proportion of developing embryos were also significantly higher in EC embryos (96.7%) compared to LC embryos (60.9%, P<0.0001).

**Fig.3:**
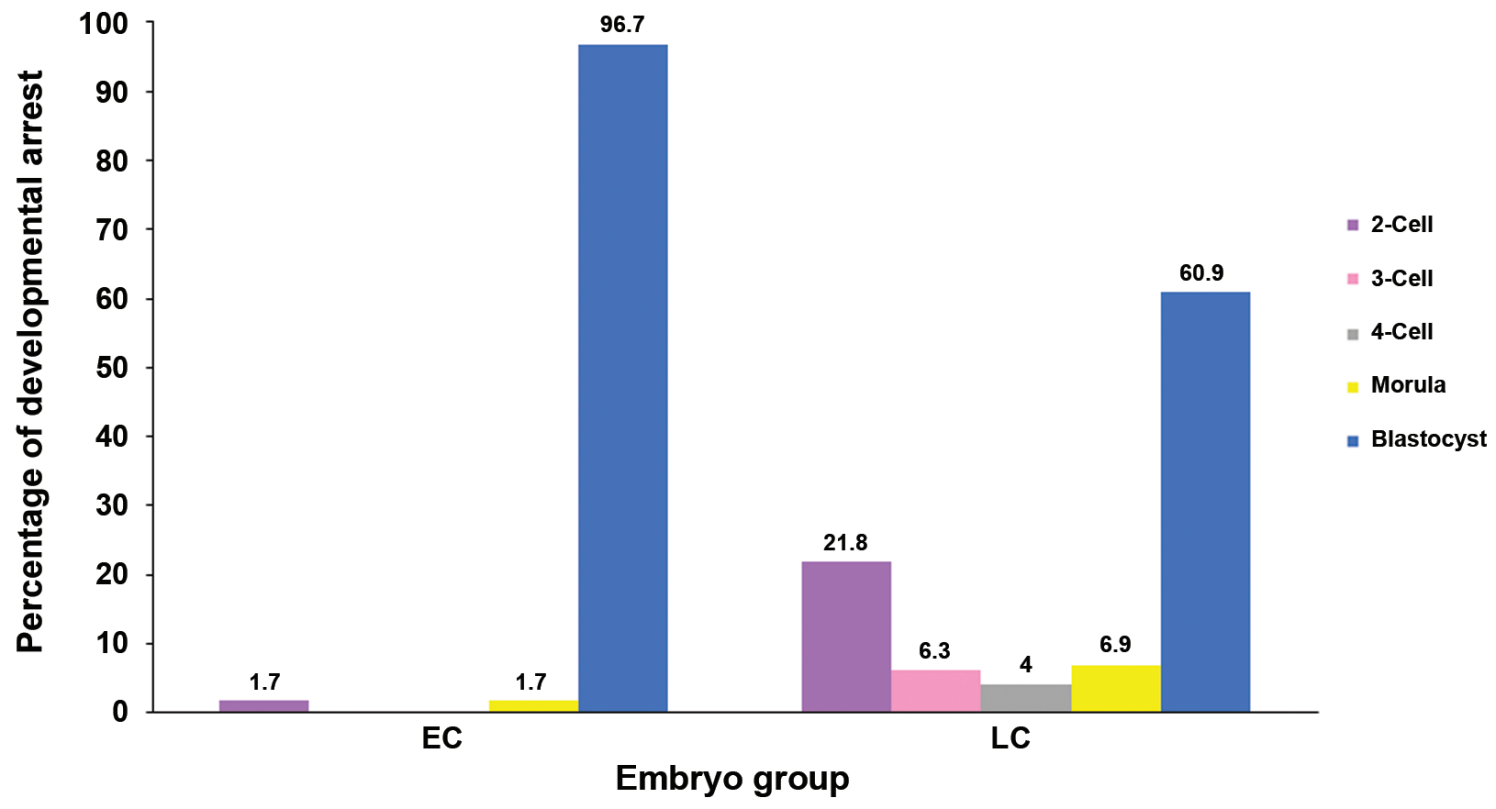
Arrest of early cleaving (EC) versus late cleaving (LC) embryos following *in vitro* culture in M16 medium for 96 hours.

The blastocyst rate was significantly higher (96.7%) for EC compared to LC embryos (60.9%, P<0.0001). The remaining EC embryos arrested at the 2-cell (1.7%) and morula (1.7%) stages. In LC embryos, besides the blastocyst stage, developmental arrest occurred at the 2-cell (21.8%), 3-cell (6.3%), 4-cell (4.0%) and morula (6.9%) stages (Fig.3).

### Experimental group (vitrified)

**Fig.4:**
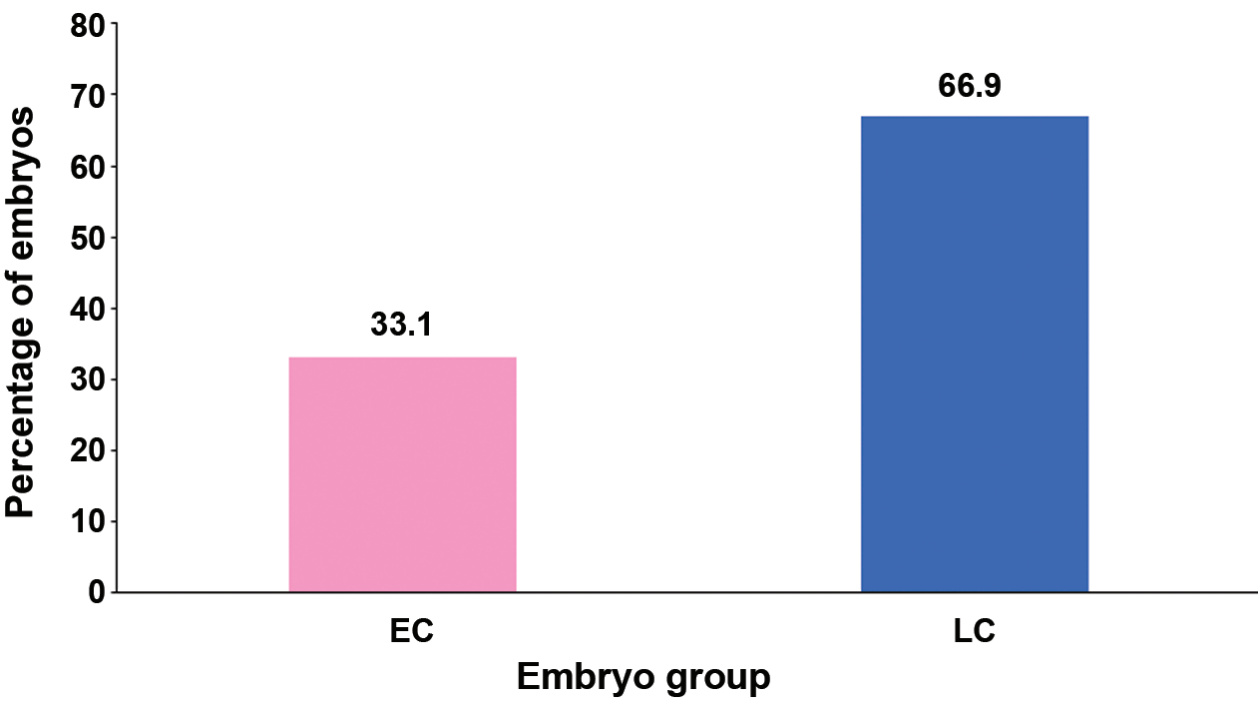
Percentage of early cleaving (EC) and late cleaving (LC) embryos from *in vivo* fertilization (treatment group).

A total of 175 embryos were included in the experimental group. Of these, 58 (33.1%) were EC embryos while the other 117 (66.9%) were LC embryos (Fig.4). Evaluation of post-vitrification survival rate showed that vitrified EC embryos demonstrated better post-vitrification survival (62.1%) than vitrified LC embryos (50.4%). However, the difference was not significant (Table 2).

After 24 hours of *in vitro* culture we observed no significant difference between vitrified EC (80.6%) and LC (71.2%) embryos. However, after longer culture (48 and 72 hours) there was a significantly higher percentage of developing embryos in the EC compared to LC embryos (P<0.05). Significantly more EC embryos reached the morula (77.8%) and blastocyst (69.4%) stages compared to LC embryos that reached the morula (42.4%) and blastocyst (27.1%) stages (P<0.0001, Table 2).

**Fig.5:**
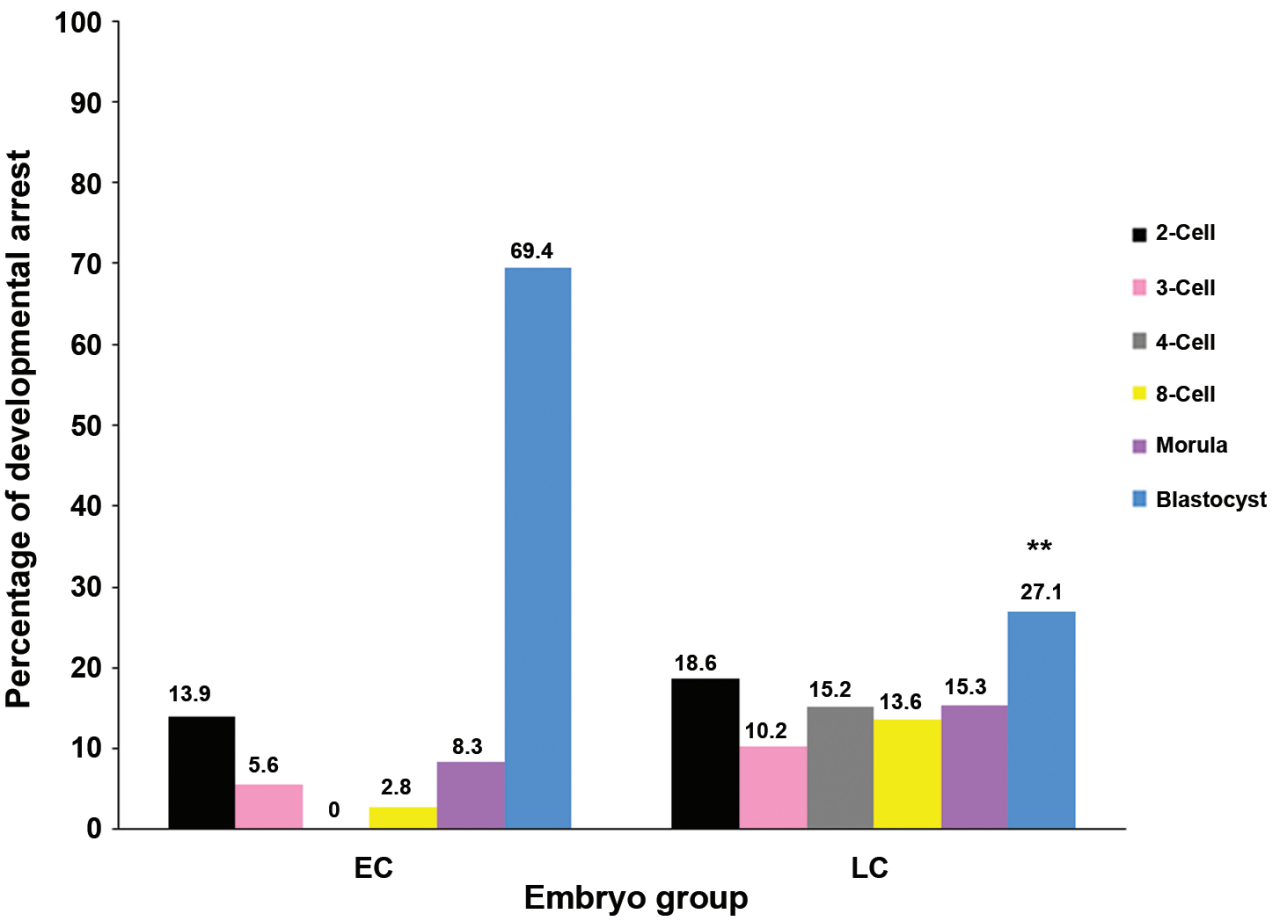
Developmental arrest of early cleaving (EC) and late cleaving (LC) embryos following vitrification and *in vitro* culture in M16 medium for 72 hours. **; P<0.0001 compared to EC embryos

The remaining EC embryos arrested at the 2-cell (13.9%), 3-cell (5.6%), 8-cell (2.8%) and morula (8.3%) stages. In LC embryos that did not reach the blastocyst stage, developmental arrest occurred at the 2-cell (18.6%), 3-cell (10.2%), 4-cell (15.2%), 8-cell (13.6%) and morula (15.3%) stages (Fig.5).

**Table 2:** Survival and developmental ability of early cleaving (EC) versus late cleaving (LC) murine embryos following vitrification and *in vitro* culture in M16 medium for 72 hours


Embryo groups	Number of embryos (%)				
	Vitrified	Survived	4-cell (24 hours post-vitrification)	Morula (48 hours post-vitrification)	Blastocyst(72 hours post-vitrification)

EC	58	36 (62.1)	29 (80.6)	28 (77.8) *	25 (69.4) **
LC	117	59 (50.4)	42 (71.2)	25 (42.4)	16 (27.1)


*; P<0.05 versus LC embryos and **; P<0.0001 versus LC embryos.

Table 3 presents a comparison of blastocyst formation between control (non-vitrified) and experimental (vitrified) groups. The percentage of blastocyst from vitrified embryos (69.4%) was significantly lower compared with non-vitrified embryos (96.7%) for EC embryos; we observed the same for vitrified (27.1%) compared to nonvitrified (60.9%) embryos among the LC embryos.

**Table 3:** Comparison of blastocyst formation between control and experimental groups following *in vitro* culture in M16 medium for 96 hours


Embryo group	Blastocyst formation, n (%)
	EC embryos LC embryos

Control (non-vitrified)	58/60 (96.7%)** 106/174 (60.9%)**
Experimental (vitrified)	25/36 (69.4%) 16/59 (27.1%)


**; P<0.0001 versus vitrified embryos

## Discussion

In order to select the best quality embryo, the embryo scoring system based on morphological assessment has been established for human ART protocols (2). However, few studies have sought to find alternative, non-invasive tools that improve selection of embryos (5, 12, 13). Timing of the first zygotic cleavage is one of the proposed parameters (5, 6, 14). As shown in humans, embryos that have cleaved early more often develop into good quality embryos with higher developmental potential (5, 6).

For human ART protocols, EC embryos are defined as those which have cleaved to the 2-cell stage at 25-27 hours after insemination (hpi) or intracytoplasmic sperm injection (ICSI) (4, 15). This corresponds to the first mitotic division. LC embryos are defined as embryos which have cleaved to the 2-cell stage >27 hpi or ICSI.

In this study, embryos were collected from oviducts of *in vivo*-fertilized mice. The results showed that under the *in vivo* conditions applied to this study, murine EC embryos displayed the 2-cell stage at 28-30 hours post-hCG administration, while LC embryos reached the 2-cell stage ≥30 hours post-hCG administration.

To our knowledge, this is the first report on the timing of the first zygotic cleavage of *in vivo*-derived embryos in a mouse model. The superiority of *in vivo*-derived mouse embryos over *in vitro*derived embryos is supported by a previous study, which has demonstrated that *in vivo*-derived bovine embryos exhibited a reduced sensitivity to chilling and freezing due to the lower lipid to protein ratio than *in vitro*-produced embryos (16).

As far as the incidence of EC is concerned, a previous study has found that this incidence ranges from 15 to 38% in humans and 32 to 76.8% in cattle (7). Whilst in the present study, we have shown the incidence of early cleavers to be 22.4% for the control group and 33.1% for the experimental group, which suggested that the percentage of early cleavers in mice was within the same range as human IVF-derived embryos.

Concerning the relationship between early cleavage status and embryo quality in the mouse, the results of the present study agreed with other studies (4, 6, 15) of humans which found that EC embryos had a significantly higher developmental potential compared to LC embryos. EC embryos observed in the present study were characterized by a significantly higher developmental rate at 24, 48, 72 and 96 hours post-hCG administration compared to LC embryos. A significantly higher percentage of developing embryos was also maintained in vitrified EC embryos compared to vitrified LC embryos in most stages, except at 48 hours of culture.

The present study revealed that EC embryos (96.7%) significantly reached the blastocyst stage compared to LC embryos (60.9%). In congruence, previous studies on porcine and human embryos also found that EC embryos had higher blastocyst formation compared to LC embryos (8, 14). Similarly, vitrified EC embryos (69.4%) showed a significantly higher percentage of blastocyst formation compared to vitrified LC embryos (27.1%). Greater cryotolerance of post-vitrification EC embryos was indicated by their better morphology, especially the intactness of the zona pellucida and blastomeres (17). This resulted in a higher developmental potential, even after exposure to high concentration of cryoprotectant and high cooling rates during the vitrification procedure.

However, the reasons for better quality and better developmental viability of EC embryos compared to LC embryos remain unknown. Whether this is related to maternal factors such as the quality of oocytes as speculated by previous studies (4, 6, 15) warrants further investigation. Lechniak et al. (7) has stated that maternal factors in oocytes have more prevalence of an impact on embryo quality than sperm, since the majority of transcripts and other cytoplasmic compounds in a zygote are of maternal origin. However, paternal factors such as the quality of spermatozoa cannot be ruled out as they contribute to the DNA of the embryos (15).

The present study also compared the blastocyst formation between control (non-vitrified) and experimental (vitrified) murine embryos. It was found that blastocyst rate from vitrified embryos was significantly lower compared with that of non-vitrified embryos i.e. 69.4 versus 96.7% for EC embryos; and 27.1 versus 60.9% for LC embryos. This is in accordance with results of a previous study on murine embryos which showed a 22.3% blastocyst rate for vitrified embryos versus 47.1% for non-vitrified embryos (18). The explanation for the decreased blastocyst rate after vitrification remains unclear. However, application of cryoprotectant at high concentrations may increase the osmolarity, which further damage the cells and destabilize cell membranes. Removal of the permeated cryoprotectant from the cell during warming may cause osmotic injuries to cells (19). All factors involved in vitrification may affect the viability of embryos and cumulatively reduce blastocyst rates.

In a study, survival rate of murine embryos following vitrification was reported to be 62% by Miyake et al. (20), whilst Uechi et al. (18) reported 77.4%. However these studies did not compare EC to LC embryos. In the present study, the survival rate of vitrified EC embryos was 62.1%, whereas for LC embryos it was 50.4%. However, there was no significant difference between the survivability of these two groups. Although no significant differences were noted, there was a consistent trend that the survived EC embryos had higher potential to develop into the blastocyst stage, compared to LC embryos.

The present research provides new information on cryotolerance of EC murine embryos. Previous studies on EC mouse embryos have concentrated more on developmental viability, blastocyst rate, pregnancy rate, implantation rate and live birth rate. There was no evidence of the quality and viability of these embryos after vitrification. Although morphological scoring of post-vitrification EC and LC at the 2-cell stage showed no significant difference, our results showed that vitrified EC embryos had higher potential to develop into blastocysts compared to vitrified LC embryos.

## Conclusion

The present study has shown that under *in vivo* conditions, EC murine embryos are superior to LC embryos in terms of post vitrification developmental viability. We suggest preselection of EC embryos as vitrification candidates for better cryopreservation outcomes to improve ART procedures.

## References

[1] Multiple gestation pregnancy (2000). The ESHRE Capri Workshop Group. Hum Reprod.

[2] Baczkowski T, Kurzawa R, Głąbowski W (2004). Methods of embryo scoring in in vitro fertilization. Reprod Biol.

[3] Fancsovits P, Toth L, Takacs F, Murber A, Papp Z, Urbancsek J (2005). Early pronuclear breakdown is a good indicator of embryo quality and viability. Fertil Steril.

[4] Fu J, Wang XJ, Wang YW, Sun J, Gemzell-Danielsson K, Sun XX (2009). The influence of early cleavage on embryo developmental potential and IVF/ICSI outcome. J Assist Reprod Genet.

[5] Nielsen HI, Ali J (2005). Embryo culture media, culture techniques and embryo selection: a tribute to Wesley Kingston Whitten. J Reprod Stem Cell Biotechnol.

[6] Lundin K, Bergh C, Hardarson T (2001). Early embryo cleavage is a strong indicator of embryo quality in human IVF. Hum Reprod.

[7] Lechniak D, Pers-Kamcyc E, Pawlak P (2008). Timing of first zygotic cleavage as a marker of developmental potential of mammalian embryo. Reprod Biol.

[8] Isom SC, Li RF, Whitworth KM, Prather RS (2012). Timing of first embryonic cleavage is a positive indicator of the in vitro developmental potential of porcine embryos derived from in vitro fertilization, somatic cell nuclear transfer and parthenogenesis. Mol Reprod Dev.

[9] Valley JK, Swinton P, Boscardin WJ, Lue TF, Rinaudo PF, Wu MC, Garcia MM (2010). Preimplantation mouse embryo selection guided by light-induced dielectrophoresis. PLoS One.

[10] Kasai M, Komi JH, Takakamo A, Tsudera H, Sakurai T, Machida T (1990). A simple method for mouse embryos cryopreservation in a low toxicity vitrification solution, without appreciable loss of viability. J Reprod Fertil.

[11] Shaw JM, Kasai M, Kola I, Tymms M (2001). Embryo cryopreservation for transgenic mouse lines. Methods in molecular biology, gene knockout protocols.

[12] Brezinova J, Oborna I, Svobodova M, Fingerova H (2009). Evaluation of day one embryo quality and IVF outcome--a comparison of two scoring systems. Reprod Biol Endocrinol.

[13] Ceyhan ST, Jackson KV, Racowsky C, Carrell DT, Racowsky C, Schlegel Pn, Van Voorhis BJ (2009). Selecting the most competent embryo. Biennial review of infertility.

[14] Van Montfoort AP, Dumoulin JC, Kester AD, Evers JL (2004). Early cleavage is a valuable addition to existing embryo selection parameters: a study using single embryo transfers. Hum Reprod.

[15] Salumets A, Hyden-Granskog C, Makinen S, Siukkari AM, Tiitinen A, Tuuri T (2003). Early cleavage predicts viability of human embryo in elective single embryo transfer procedures. Hum Reprod.

[16] Leibo SP, Loskutoff M (1993). Cryobiology of in vitro-derived bovine embryos. Theriogenology.

[17] Zhang J, Cui J, Ling X, Li X, Peng Y, Guo X (2009). Vitrification of mouse embryos at 2-cell, 4-cell and 8-cell stages by cryotop method. J Assist Reprod Genet.

[18] Uechi H, Tsutsumi O, Morita Y, Takai Y, Taketani Y (1999). Comparison of the effects of controlled-rate cryopreservation and vitrification on 2-cell mouse embryos and their subsequent development. Hum Reprod.

[19] Nagy ZP, Vajta G, Chang CC, Kort H, Gardner DK, Weissman A, Howles CL, Shoham Z (2009). The human embryo: vitrification. Textbook of assisted reproductive technologies laboratory and clinical perspectives.

[20] Miyake T, Kasai M, Zhu SE, Sakurai T, Machida T (1993). Vitrification of mouse oocytes and embryos at various stages of development in an ethylene glycol-based solution by a simple method. Theriogenology.

